# The Olfactory Chemosensation of Hematophagous Hemipteran Insects

**DOI:** 10.3389/fphys.2021.703768

**Published:** 2021-08-09

**Authors:** Feng Liu, Zhou Chen, Zi Ye, Nannan Liu

**Affiliations:** ^1^Department of Entomology and Plant Pathology, Auburn University, Auburn, AL, United States; ^2^Department of Biological Sciences, Vanderbilt University, Nashville, TN, United States; ^3^Cardiovascular Research Institute, University of California, San Francisco, San Francisco, CA, United States

**Keywords:** bed bug, kissing bug, host-seeking behavior, peripheral olfactory system, olfaction, push-pull strategies, reverse chemical ecology

## Abstract

As one of the most abundant insect orders on earth, most Hemipteran insects are phytophagous, with the few hematophagous exceptions falling into two families: Cimicidae, such as bed bugs, and Reduviidae, such as kissing bugs. Many of these blood-feeding hemipteran insects are known to be realistic or potential disease vectors, presenting both physical and psychological risks for public health. Considerable researches into the interactions between hemipteran insects such as kissing bugs and bed bugs and their human hosts have revealed important information that deepens our understanding of their chemical ecology and olfactory physiology. Sensory mechanisms in the peripheral olfactory system of both insects have now been characterized, with a particular emphasis on their olfactory sensory neurons and odorant receptors. This review summarizes the findings of recent studies of both kissing bugs (including *Rhodnius prolixus* and *Triatoma infestans*) and bed bugs (*Cimex lectularius*), focusing on their chemical ecology and peripheral olfactory systems. Potential chemosensation-based applications for the management of these Hemipteran insect vectors are also discussed.

## Introduction

The insect order Hemiptera, one of the most abundant insect orders, encompasses a wide range of different species. Although most hemipteran insects feed on plants or other insects, small invertebrates or even sugars (Díaz-Albiter et al., 2016), a few, such as kissing bugs and bed bugs, utilize blood sources from humans and/or animals [for more details, see the review provided in Reinhardt and Siva-Jothy (2007)]. Bed bugs (Cimicidae) have been reported to be resurgent in many developed countries due to the relaxation of monitoring systems, the development of insecticide resistance, and the increase in international travel in recent years (Doggett et al., 2004, 2012; Ter Poorten and Prose, 2005; Romero et al., 2007; Yoon et al., 2008; Haynes and Potter, 2013; Zhu et al., 2013). Kissing bugs, which are members of the *Triatominae* subfamily of the family Reduviidae, are typically found in the southern United States, Mexico, Central America, and South America (Justi et al., 2016; Monteiro et al., 2018).

Both kissing bugs and bed bugs are obligate blood-feeding ectoparasites of multiple hosts, including mammals, birds, and reptiles. For human beings, the major concerns related to these two hemipteran insects lie in their biting nuisance and their potential role as disease vectors. Bites from bed bugs result in the victims experiencing clinical symptoms such as a wheal-and-flare response, infiltrated papules, vesicles, and/or blisters (Sansom et al., 1992; Alexander, 1994). In addition to the biting nuisance, bacterial infections such as impetigo, ecthyma, cellulitis, and lymphangitis may occur (Burnett et al., 1986). Another concern is the potential vector capacity of bed bugs. A preliminary study suggested that bed bugs probably share the same role as kissing bugs in transmitting *Trypanosoma cruzi*, the flagellate protozoan responsible for American trypanosomiasis, which is better known as Chagas disease. Using mice as their animal model, Salazar et al. (2014) found bed bugs to be a competent vector of *T. cruzi* and that they were able to efficiently and bi-directionally transmit *T. cruzi* to host mice. Most of the bed bugs fed on experimentally infected mice acquired the parasites, and a majority of the previously uninfected mice became infected after cohabitating with the exposed bed bugs in a laboratory environment. *T. cruzi* was also transmitted to mice who were directly exposed to the feces of infected bed bugs. Blakely et al. (2018) found live *T. cruzi* in the gut contents of bed bug adults fed with *T. cruzi*-contaminated blood and this persisted for at least 97 days post-infection in adult bed bugs. More importantly, they also found that nymphal stage bed bugs that were infected with *T. cruzi* maintained the parasite after molting, indicating the capacity for transstadial passage of *T. cruzi* in bed bugs.

As with bed bugs, the reaction to a kissing bug bite depends on the victim’s sensitivity toward the substances introduced during the biting process. A typical light reaction to the kissing bug bite is papular lesions with a central punctum or grouped small vesicles; severe symptoms can include giant urticarial-type lesions with swelling at the site of inoculation; hemorrhagic nodular-to-bullous lesions; conjunctivitis, and a generalized morbilliform eruption (Shields and Walsh, 1956; Hemmige et al., 2012). Kissing bugs are known to be the primary vector of the pathogen *T. cruzi* (Stevens et al., 2011; Lidani et al., 2019). Surveys conducted in the United States have indicated that about half of the *Triatominae* species identified were carrying *T. cruzi* (Davis et al., 1943). Two of the epidemiologically important vectors are *Rhodnius prolixus* Stal and *Triatoma infestans* Klug (Coura, 2015). However, unlike the transmission cycle reported for bed bugs, *T. cruzi* is transmitted by kissing bug through various manners, including vector feces, food contamination, blood transfusion, of which oral transmission by food contamination plays the major role (Pereira et al., 2010; Shikanai-Yasuda and Carvalho, 2012).

As both kissing bugs and bed bugs pose a significant risk to humans and are thus a major concern for public health, remarkable progress has been made in recent decades in elucidating their chemical ecology and olfactory physiology. This review focuses on recent advances in: 1) the factors that regulate the host-seeking behavior of bed bugs and kissing bugs; 2) the mechanisms of peripheral chemosensory system in kissing bug and bed bug, including olfactory sensilla, olfactory receptor neurons (ORNs), odorant binding proteins (OBPs) and chemosensory proteins (CSPs), odorant receptors (ORs), ionotropic receptors (IRs), and gustatory receptors (GRs); and 3) perspectives for chemosensation-based applications in the management of kissing bug and bed bugs. This emerging knowledge is expected to make a positive contribution to the control of these blood-feeding insects and thus reduce the potential disease transmissions.

### Host-Seeking Behavior of Kissing Bugs and Bed Bugs

Since both kissing bugs and bed bugs rely on human or animal blood sources for survival and reproduction, host localization is a vital part of their daily activities. In the host-seeking process, heat, host odor, and carbon dioxide (CO_2_) are important cues for both kissing bugs and bed bugs. Kissing bugs (*R. prolixus* and/or *T. infestans*) were found to be attracted to warm temperature (Wigglesworth and Gillett, 1934; Milne et al., 2009), host-related compounds (Bodin et al., 2009; Milne et al., 2009; Ortiz and Molina, 2010; Ortiz et al., 2011), and CO_2_ (Wiesinger, 1956; Nunez, 1982; Guerenstein and Guerin, 2001; Barrozo and Lazzari, 2004; Guerenstein and Lazarri, 2009; Indacochea et al., 2017). Kissing bug nymphs are attracted by CO_2_-free traps baited with three host-odor components (ammonia, L-(+)-lactic acid, and hexanoic acid) but not by traps containing either one component alone or two components, suggesting a synergistic effect of host odors in attracting kissing bugs (Guidobaldi and Guerenstein, 2013). Researchers have also found that bed bugs can distinguish temperature differences as low as 1–2°C *via* the thermosensors on their antennae (Sioli, 1937). Heat baited traps attract significantly more bed bugs than unheated traps (Wang et al., 2009; Anderson et al., 2017). CO_2_ baited traps are also more attractive for bed bugs than non-CO_2_ traps and CO_2_ are more effective than heat in trapping assays (Wang et al., 2009). In addition, bed bugs respond to human skin swabs in the absence of all other host cues (DeVries et al., 2019). However, chemical lures baited with specific human odors displayed more complex results, with the trapping efficiency largely depending on the specific compounds incorporated into the lures. For instance, Wang et al. (2009) found that lures baited with two human odors, 1-octen-3-ol, and L-lactic acid, did not attract significantly more bed bugs than non-baited traps, while Anderson et al. (2017) reported that ammonium bicarbonate and a blend of (E)-2-hexenal and (E)-2-octenal at certain concentrations attracted more bed bugs than the untreated control. In another study, Singh et al. (2012) screened twelve chemicals, evaluated the interactions among chemical lures, CO_2_, and heat in trapping bed bugs, and revealed a synergistic effect between chemical lures and CO_2_ but not heat and CO_2_.

Multiple factors have been determined to regulate the host-search activities of both kissing bugs and bed bugs, including food source availability, mating status, and temporal modulation. Studies have shown that starvation plays a critical role in affecting the olfactory responses of kissing bugs (*R. prolixus*) to host odors, with starved *R. prolixus* showing a significant preference for the host-odorant treated arm in a dual-choice olfactometer, while a random distribution was observed in non-starved kissing bugs (Reisenman et al., 2013). Similarly, bed bugs that have been starved for a week were found to be more active in host-searching than those that had received a blood meal 2 days before testing (Romero et al., 2010). Bed bugs that have received a blood meal are also more likely to aggregate in shelters during the scotophase, while those that have not fed tend to spend more time out of the shelters (Reis and Miller, 2011).

Another factor in determining bed bugs’ host-searching activities is mating status. The percentage of females that fed and the amount of blood they ingested were found to be significantly greater in mated females than in unmated females and far more mated than unmated females responded to human odors (DeVries et al., 2019; Saveer et al., 2021). Interestingly, starvation also has a strong impact on the response of mated or unmated female bed bugs to human odors. The response rate of unmated females to skin odor increased with longer starvation periods, while the opposite pattern was observed in mated females (Saveer et al., 2021). Temporal modulation also plays a critical role in determining host-seeking activity. Behavior-related antennal sensitivity is governed by a circadian clock or daily rhythm in multiple insect species, including moths, flies, cockroaches, bed bugs, and kissing bugs (Brady, 1975; Hawkins and Rust, 1977; Van der Goes van Naters et al., 1998; Krishnan et al., 1999; Page and Koelling, 2003; Rosén et al., 2003; Bodin et al., 2008). An endogenous circadian clock has also been found to affect the insect’s orientation toward CO_2_, but only during the scotophase for both *T. infestans* and *R. prolixus* (Barrozo et al., 2004; Barrozo and Lazzari, 2004; Bodin et al., 2008). In addition, Reisenman (2014) reported that the electroantennogram (EAG) response of starved *R. prolixus* to ammonia (a host odor) was significantly higher than in insects fed only during the night. This modulation of sensory responses at the neural level is believed to trigger host search behavior in starved kissing bugs. In bed bugs, their spontaneous locomotor activity is known to be determined by an inner circadian rhythm, with both adults and nymphs being much more active in the dark than in the light phase (Romero et al., 2010). This is thought to enhance their chance of locating a sleeping human host (Romero et al., 2010).

### Mechanism of Peripheral Olfactory System

**Figure 1:**
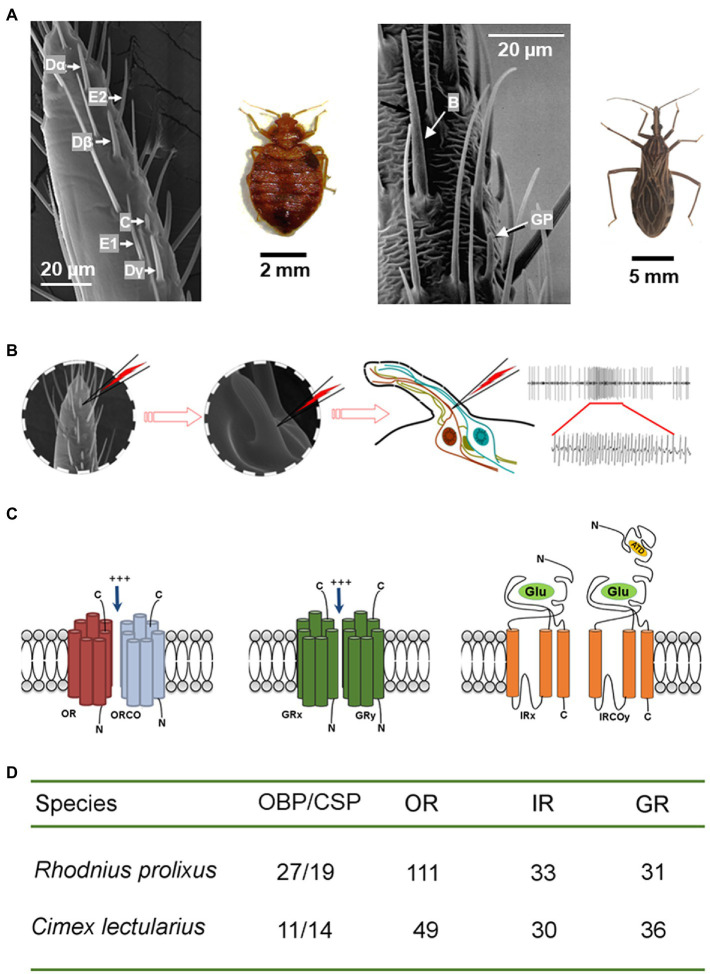
Olfactory mechanism of the peripheral olfactory system in bed bugs and kissing bugs. **(A)** Scanning electronic microscope images show six functional types of olfactory sensillum (Dα, Dβ, Dγ, E1, E2, and C) for bed bugs (left; Liu and Liu, 2015) and two types (Basiconica and grooved peg) for kissing bugs (right; adapted from Guerenstein and Guerin, 2001, with the permission from Dr. Guerin). **(B)** The olfactory receptor neurons housed in each olfactory sensillum are responsible for detecting the attractive cues and increasing the firing frequency of the action potentials. *Left*: one section of a bed bug antennae; *middle*: a single sensillum is shown at high magnification (x720); right: depiction showing that the recording tungsten electrode is inserted into the shaft of a sensillum to complete the electrical circuit and to extracellularly record the olfactory receptor neuron potentials. **(C)** Schematic diagrams of the structures of three olfactory receptors (OR/ORCO, GR, and IR/IRCO) expressed in the membranes of the neuron dendrites that are the molecular targets for host cues. **(D)** The total number of odorant binding proteins and olfactory receptors (OR/ORCO, GR, and IR/IRCO) identified in the genomes of *C. lectularius* and *R. prolixus*.

Kissing bugs and bed bugs, like other insects, sense their chemical environment through their peripheral olfactory system. Their major olfactory appendages are their antennae, where various morphological or functional types of olfactory sensilla are located (Figures 1A,B). Olfactory sensory neurons (OSNs) are housed in each olfactory sensillum and OBPs/CSPs are secreted into the sensillum lymph by the accessory cells. Specific or unique olfactory receptors, including ORs, IRs, and CO_2_-specific GRs, are expressed on the membrane of these OSNs (Figure 1C). Odorants surrounding the antennae pass through the pores on the sensillum surface and potentially bind with the OBPs/CSPs, after which they are delivered to active sites on the olfactory receptors (Brito et al., 2016). When olfactory receptors are activated by specific ligands, the cation channel formed by the olfactory receptors will be open (Nakagawa et al., 2005; Sato et al., 2008), which leads to the depolarization of OSNs and generation of action potentials. The chemical information is then transformed into electrical signals in the OSNs and transmitted along the axons into the antennal lobe in the central nervous system, where chemical information is further processed before the final behavioral decisions are made (Carey and Carlson, 2011; Leal, 2013). While the peripheral olfactory system of kissing bug is comparable with other blood-feeding insects (e.g. mosquito *Anopheles gambiae*) in term of the amount of olfactory sensilla and ORs, bed bugs are found to possess a degenerative olfactory system with much fewer olfactory sensilla and ORs (Levinson et al., 1974; Benoit et al., 2016).

#### Olfactory Sensilla and Olfactory Receptor Neurons

The olfactory sensilla make up a key structure that plays a critical role in the chemosensation of the insect antennae. Based on their morphological shape, the common bed bug (*C. lectularius*) has three types of olfactory sensilla: D, C, and E (Table 1). Of these, the majority are distributed along the distal portion of flagellomere II, with just a few located in the pedicel (Harraca et al., 2010; Liu et al., 2013; Olson et al., 2014). Each different type of sensillum houses a varying number of neurons. Three functional types of D sensilla (D*α*, D*β*, D*γ*), two types of C sensillum (C1, C2), and two E sensilla (E1, E2) have been identified on flagellomere II. A refined distribution map for each type of sensillum was described by Liu et al. (2017c). D*α*, D*β*, D*γ*, C1, C2, E1, and E2 have all been identified as olfactory sensilla, while the third type of E sensillum (E3) is thought to be a gustatory sensillum (Singh et al., 1996; Olson et al., 2014). The numbers of olfactory sensilla presenting on the antenna gradually increase as *C. lectularius* progresses from the first nymph instar to the adult stage, but no sexual dimorphism has been observed in either the sensillum number or their distribution along the antenna (Liu et al., 2017c). This is also the case for another bed bug species, the tropical bed bug (*C. hemipterus*), where the number of chemo-sensilla (olfactory and gustatory sensilla) on the antenna again increase from the nymph to the adult stage, with no sexual dimorphism (Mendki et al., 2013). However, the chemo-sensilla are distributed across all four segments of the antennae in the tropical bed bugs, while no chemo-sensilla have been found in either the base or the flagellomere I of the common bed bug antenna (Mendki et al., 2013; Olson et al., 2014). There are also reports of a few chemo-sensilla being seen in the rostrum of the tropical bed bug but not in the common bed bug (Mendki et al., 2013).

**Table 1:** Types and functions of the antennal sensilla in the common bed bug (Cimicidae) and kissing bug (Triatominae).

Sensillum	Type	Distribution	Number	Number of neurons	Function	References^*^
Cimicidae (common bed bug)	D	flagellomere II	6	8–19	Chemoreceptors (Olfaction)	[1–8]
	C	flagellomere II	9	4–5	Chemoreceptors (Olfaction)	
	E	Pedicel flagellomere II	34	1–3	Chemoreceptor (Olfaction and gustation)	
Triatominae (kissing bug)	Trichoidea	Pedicel flagellomere I/II	200–800	5–15	Chemoreceptors	[9–24]
	Basiconica	flagellomere I/II	40–130	5–6	Chemoreceptors and thermohygroreceptors	
	Coeloconica	Pedicel flagellomere I/II	5	3	Thermohygroreceptors	
	Cave organ	Pedicel	1	200–300	Thermoreceptors	

* [1] Levinson et al., 1974; [2] Steinbrecht and Müller, 1976; [3] Singh et al., 1996; [4] Harraca et al., 2010; [5] Liedtke et al., 2011; [6] Olson et al., 2014; [7] Liu et al., 2014; [8] Liu and Liu, 2015; [9] Wigglesworth and Gillett, 1934; [10] Mayer, 1968; [11] Bernard, 1974; [12] Mciver and Siemicki, 1985; [13] Lazzari, 1990; [14] Catalá and Schofield, 1994; [15] Lazzari and Wicklein, 1994; [16] Catalá, 1997; [17] Taneja and Guerin, 1997; [18] Guerenstein and Guerin, 2001; [19] Carbajal De La Fuente and Catalá, 2002; [20] Diehl et al., 2003; [21] Catalá et al., 2004; [22] Villela et al., 2005; [23] Moreno et al., 2006; [24] Pontes et al., 2014.

In kissing bugs, four morphological types of sensillum have been characterized in the antenna, namely trichoidea, basiconica, coeloconica, and cave organ (Table 1; Barrozo et al., 2017). Trichoidea and basiconica are the most common types on flagellomeres I and II, both of which function as chemoreceptors. Two subtypes of trichoidea, multi- and uni-porous, have been identified based on the number of pores on individual sensilla (Guidobaldi et al., 2014). Multi-porous trichoidea sensilla sense odors, whereas uni-porous sensilla (with a single pore at the tip) detect tastants (Mayer, 1968; Taneja and Guerin, 1997; Guerenstein and Guerin, 2001; Diehl et al., 2003; Pontes et al., 2014). Sensilla coeloconica are assumed to perform a thermohygrom receptive function in *Triatominae*; basiconica may also perform the same function (Bernard, 1974; Mciver and Siemicki, 1985; Lazzari, 1990). Only one cave-like sense organ has been found on the pedicel segment and electrophysiological evidence supports a thermoreceptive role for this organ (Catalá and Schofield, 1994; Lazzari and Wicklein, 1994).

The distribution of sensory organs on triatomine antennae displays a genus-, sex-, and habitation-biased pattern. For example, the total number of trichoidea sensilla varies dramatically between *Triatoma* (400–800) and *Rhodnius* (200–500; Catalá and Dujardin, 2001; Carbajal De La Fuente and Catalá, 2002; Catalá et al., 2004, 2005; Esteban et al., 2005; Villela et al., 2005; Moreno et al., 2006; Carbajal De La Fuente et al., 2008; Villacís et al., 2010; May-Concha et al., 2016). *Triatoma* males have trichoidea sensilla that are significantly more thin-walled than those of the females, especially on the pedicel segment (Catalá et al., 2004; Villela et al., 2005; May-Concha et al., 2016), whilst the number of thin-walled trichoidea sensilla in the *Rhodnius* species exhibit no difference between the sexes (Catalá et al., 2004; Villacís et al., 2010). Interestingly, *T. infestans* collected from domestic sites have more thin-walled trichoidea sensilla on the pedicel and more thick-walled trichoidea sensillum on both flagellomere I and II than those collected from sylvan sites (Catalá and Dujardin, 2001; Catalá and Torres, 2001) with the specific mechanism yet to be determined.

**Figure 2:**
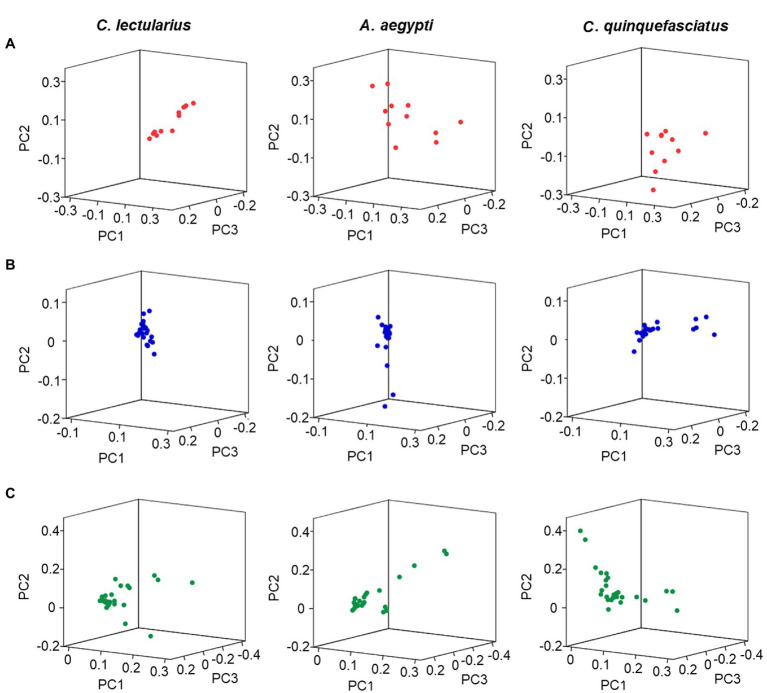
Distribution of odorants in an ORN activity-based odor space. Odor spaces were constructed using the first three principal components of PCA (PAST 3.0, Carey et al., 2010) for the primary sensory responses generated by odorants (Liu et al., 2013, 2014; Liu and Liu, 2015; Ye et al., 2016; Chen et al., 2018, 2019). **(A)** Aldehydes; **(B)** aromatics; **(C)** terpenoids. All odorants from each chemical class are included. The mean inter-odorant distances in three-dimensional space for the set of aldehydes are 0.16 ± 0.01 for *C. lectularius*, 0.34 ± 0.02 for *A. aegypti*, and 0.22 ± 0.01 for *C. quinquefasciatus* (*A. aegypti* vs. *C. lectularius*, *p* < 0.001; *C. quinquefasciatus* vs. *C. lectularius*, *p <* 0.001, *t*-test). The mean inter-odorant distances for aromatics are 0.05 ± 0.00 for *C. lectularius*, 0.10 ± 0.01 for *A. aegypti*, and 0.10 ± 0.01 for *C. quinquefasciatus* (*A. aegypti* vs. *C. lectularius*, *p* < 0.001; *C. quinquefasciatus* vs. *C. lectularius*, *p* < 0.001, *t*-test). The mean inter-odorant distances for terpenoids are 0.11 ± 0.01 for *C. lectularius*, 0.14 ± 0.01 for *A. aegypti*, and 0.16 ± 0.01 for *C. quinquefasciatus* (*A. aegypti* vs. *C. lectularius*, *p* < 0.001; *C. quinquefasciatus* vs. *C. lectularius*, *p* < 0.001, *t*-test).

Potent sensitivities of the kissing bug olfactory sensillum to host odor plumes and a few unitary aldehyde and acid compounds have been described (Guerenstein and Guerin, 2001), while the bed bug olfactory sensilla are particularly sensitive to several chemical classes of odors in human emanation, especially aldehydes, alcohols, aromatics, and ketones (Liu and Liu, 2015), as well as plant-sourced terpenes and terpenoids (Liu et al., 2014). Similar patterns have also been reported for two mosquitoes, *Culex quinquefasciatus* and *Aedes aegypti* (Liu et al., 2013; Ye et al., 2016; Chen et al., 2018, 2019). As bed bugs possess far fewer olfactory sensilla/OSNs than either kissing bugs or mosquitoes, their capacity for odor discrimination is likely to be inferior. Indeed, a comparison of the distribution of multiple groups of compounds in the odor space of bed bugs, *C. quinquefasciatus* and *A. aegypti* indicates that bed bugs may be less capable of discriminating human-related aldehydes and aromatics and plant-related terpenoids than either *Culex* or *Aedes* mosquitoes (Figure 2). These differences in odor-discriminatory capacity probably lie in the much more abundant functional types of olfactory sensilla or OSNs in the antenna of *C. quinquefasciatus* and *A. aegypti* compared to bed bugs. Although as yet there is insufficient data to include kissing bugs in this comparison, it is reasonable to speculate that kissing bugs are likely to be endowed with a much stronger ability for odor discrimination than bed bugs as they have a comparable number of olfactory sensilla to mosquitoes and live in a similarly complex chemical environment.

#### Odorant-Binding Proteins and Chemosensory Proteins

Odorant-binding proteins (OBPs) and CSPs, low-molecular-weight soluble proteins that are secreted by the accessory cells, are highly concentrated in sensillum lymph. OBPs and CSPs function to transport hydrophobic odorants through the aqueous environment of the sensillum lymph to the ORs’ recognition sites. According to the various models that have been proposed, an OR may be activated either by the odorant molecule itself or the OBP(CSP)/odorant complex (Leal, 2013). For instance, knockdown of OBP1 in the southern house mosquito *C. quinquefasciatus* results in reduced EAG responses to mosquito oviposition pheromones (Pelletier et al., 2010) and silencing OBP1 leads to a failure to sense indole, a key component of human sweat, in the malaria mosquito *Anopheles gambiae* (Biessmann et al., 2010). In the tsetse fly, silencing the OBPs that interact with 1-octen-3-ol dramatically abolished flies’ attraction to 1-octen-3-ol (Diallo et al., 2021), while in brown planthopper, silencing one CSP gene (*NlugCSP8*) induced significant decrease in the behavioral responses to some representative attractants (Waris et al., 2018). With many studies suggesting the essential roles of OBPs and CSPs in the chemosensation of some insect species, there are also opposite discoveries about the odor-transporting role of the OBPs (or CSPs). For example, it is also reported that a fly strain with all *obp* genes deleted still showed robust responses to odors from diverse chemical groups (Xiao et al., 2019), which suggests other functions of OBPs or CSPs beyond odor transportation in the olfactory sensillum. Actually, only a small number of OBPs or CSPs have been found in the olfactory appendages of various insects and some are expressed in non-sensory tissues such as sex pheromone glands of Lepidoptera, venom glands of wasps, and reproductive organs (Dippel et al., 2014; Brito et al., 2016; Sun et al., 2018), which are thus assumed to function as a carrier of internal chemicals other than external compounds (Pelosi et al., 2014). Other potential roles of OBPs or CSPs, such as contributing to the selectivity of odorant sensation or acting as odorant-degrading enzymes, have also been proposed but remain to be confirmed (Leal, 2013; Larter et al., 2016; Scheuermann and Smith, 2019).

Genome sequencing has contributed greatly to research in this area, which identifies 11 OBPs and 14 CSPs in the common bed bug (*C. lectularius*) and 27 OBPs and 19 CSPs in kissing bugs (*R. prolixus*; Figure 1D; Mesquita et al., 2015; Benoit et al., 2016). Transcriptome sequencing of olfactory appendages (antennae or rostrum) in another kissing bug species, *Triatoma brasiliensis*, also identified 27 OBPs and 17 CSPs, most of which have well-supported orthologs in *R. prolixus* (Marchant et al., 2016). Proteomic analysis of the antenna of *R. prolixus* by Oliveira et al. (2017) identified 17 OBPs and 6 CSPs, representing 63 and 31% of all the OBPs and CSPs, respectively, in the genome sequence (Mesquita et al., 2015). Further work by Oliveira et al. (2018) indicated that of the 17 OBP genes identified in the *R. prolixus* adults, although 11 were expressed in all tissues, six were specific to antennae. Of the six antenna-expressing OBPs, two (*RproOBP6* and *RproOBP13*) were expressed in both sexes; two (*RproOBP17* and *RproOBP21*) were female antenna-enriched, and the rest (*RproOBP26* and *RproOBP27*) were male antenna-specific. *RproOBP27* was later confirmed to be involved in the detection of sex pheromones by functional studies (Oliveira et al., 2018). For bed bugs, the functions of OBPs and CSPs have not yet been explored. Given that multiple experimental approaches including RNA interference (Pelletier et al., 2010), CRISPR/Cas9 (Scheuermann and Smith, 2019; Xiao et al., 2019), and competitive binding assays using a fluorescent probe (Brito et al., 2016) have been successfully used to investigate the function of OBPs or CSPs from many other insect species, future studies using similar approaches should yield interesting results about the interactions between bed bug or kissing bug OBPs or CSPs and a wide variety of biologically relevant compounds that have been examined in either electrophysiological or behavioral studies. X-ray crystallography and nuclear magnetic resonance (NMR) are other powerful tools that can provide more details about the unbound or the agonist/antagonist-bound structural complex (Brito et al., 2016), Comparison of the unbound and ligand-bound OBP structures should help identify the amino acid residues involved in ligand binding. All these valuable information will help build our understanding of the mechanisms through which compounds are filtered and transported in the sensillum.

#### Odorant Receptors

**Figure 3:**
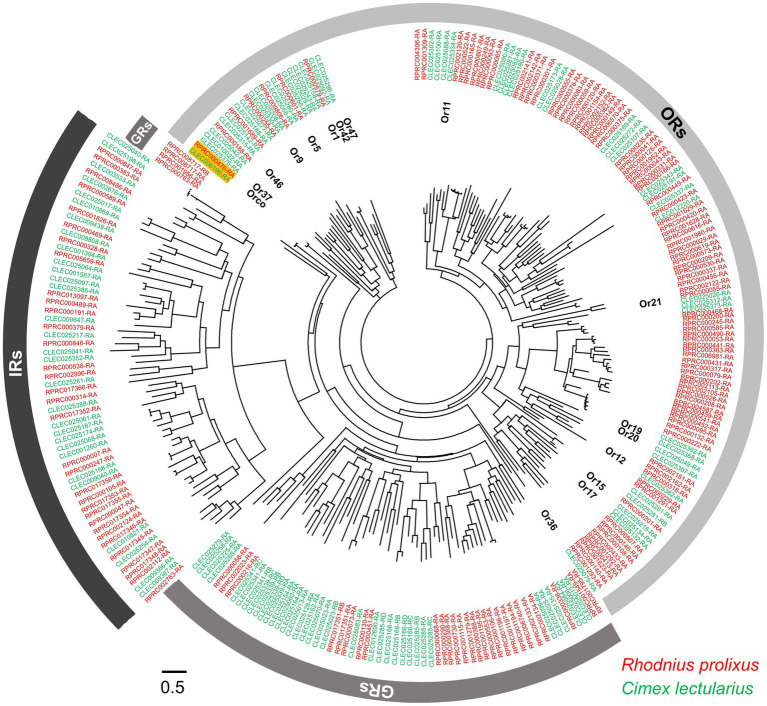
Phylogenetic relationships within the ORs, IRs, and GRs of *R. prolixus* and *C. lectularius*. The dendrogram was computed using FastTree based on a MAFFT alignment of 272 amino acid sequences (VectorBase) from *R. prolixus* (accession number in red color) *and C. lectularius* (accession number in green color). The accession numbers of two *Orco* genes are highlighted. Fifteen ORs of *C. lectularius* that have been tested against around 150 odorants, including human odors and botanical chemical stimuli, are annotated (Liu et al., 2017a).

Odorant receptors (ORs) have been extensively studied due to their role in detecting odors from diverse chemical groups (Carey et al., 2010; Joseph and Carlson, 2015; McBride, 2016; Liu et al., 2017a). ORs may evolve from IRs/GRs and are further diversified phylogenetically across different insect taxa (Hansson and Stensmyr, 2011; Missbach et al., 2014; Figure 3). However, the odorant receptor co-receptor (Orco) gene is highly conserved across insects (Jones et al., 2005; Leal, 2013). The ORCO protein is considered to play an important role in 1) the localization and stabilization of ORs in the neuron dendritic membranes; and 2) the transient binding and transduction of odorants *via* a heteromeric OR/ORCO complex (Larsson et al., 2004; Benton et al., 2006; see also the review in Stengl and Funk, 2013). Studies on the *Orco* gene of the kissing bug (*RproOrco*) revealed that when it has been silenced by RNA interference, the kissing bug is unable to locate a vertebrate host in a timely manner, leading to decreased blood ingestion, delayed and decreased molt rate, increased mortality rate, and decreased egg-laying (Franco et al., 2016). The expression level of the *RproOrco* gene is regulated by both the kissing bug’s feeding status and developmental stage. A significant decrease in *RproOrco* expression has been observed after blood feeding, while an increase follows an imaginal molt (Latorre-Estivalis et al., 2015). In the common bed bug, the *Orco* gene has been found in both olfactory appendages (antennae and legs) and other non-olfactory related tissues (Hansen et al., 2014). Interestingly, phylogenetic analysis has indicated that *R. prolixus* and *C. lectularius Orco* are closely related, with a relatively close evolutionary distance compared to other insect species in different orders (Liu and Liu, 2015).

Whole-genome sequence analyses have revealed 115 and 49 ORs for *R. prolixus* and *C. lectularius*, respectively (Figures 1D; Mesquita et al., 2015; Benoit et al., 2016). The striking difference in OR number between these two hemipteran species is thought to be correlated with the complexity of the chemical environment in their respective habitats. The wingless *C. lectularius* lives in relatively closed and limited spaces, indoors or near the host, while the winged *R. prolixus* can fly long distances for host/mate searching (Gringorten and Friend, 1979; Zacharias et al., 2010). This natural selection may result in a comparatively stable chemosensory ecology in *C. lectularius*, which presents rare *OR* gene expansion in the genome compared to *R. prolixus* (Liu et al., 2017a). Benefiting from the availability of the genomic information for these species, the expression patterns for some of the *OR*s in *R. prolixus* have been characterized for different tissues and developmental stages. Using RT-PCR, Latorre-Estivalis et al. (2016) discovered that the *R. prolixus OR*s were expressed in every development stage from embryo to nymph and adult antennae. Most of these *OR*s were found not only in the antennae but also in other tissues such as the rostri, tarsi, tibial pads, and genitalia, suggesting that these appendages may also involve in the chemosensation-mediated behaviors of *R. prolixus*. Similarly, the *ORs* in *C. lectularius* have also been found to be expressed in other structures (e.g. legs) in addition to antennae (Liu and Liu, 2015).

Functional studies aimed at deciphering insect ORs generally use one of the following experimental approaches: 1) *Drosophila* “Empty Neuron” transgenic system, where exogenous OR genes are expressed in certain fly ORNs without the expression of any native ORs (Hallem and Carlson, 2006); 2) neuron-specific calcium imaging, which monitors calcium activity in GCaMP-expressed tissues or organs, mostly in flies and mosquitoes (Silbering and Galizia, 2007); 3) *Xenopus* oocyte expression systems, which are coupled with a two-electrode voltage clamp/patch clamp to detect the receptor current through the ion channels on oocyte membrane (Wang et al., 2010); 4) mammalian cell expression system coupled with patch clamp to measure the receptor current and ion conductance of the channels (Jones et al., 2011); 5) chemical informatics, which utilizes *in silico* modeling to screen large chemical space and identify potential ligands for receptors (Boyle et al., 2013); and 6) gene editing-mediated mutagenesis, which uses gene editing techniques such as clustered regularly interspaced short palindromic repeats/ CRISPR-associated protein 9 (CRISPR/Cas9), Transcription activator-like effector nucleases (TALEN), or zinc-finger nuclease (ZFN) to create mutants and then compares the phenotype changes between the wildtype and mutant insects (McMeniman et al., 2014; Liu et al., 2021). For example, functional studies have been used to investigate the role of four kissing bug ORs in perceiving sex pheromones using a *Xenopus* oocyte expression system coupled with a two-electrode voltage clamp (Franco et al., 2018). Although none of these ORs were identified as sex pheromone receptors, RproOR80 was found to be extremely sensitive to several compounds that turned out to be repellents for kissing bugs (Franco et al., 2018). In the common bed bug, 15 ORs have been successfully expressed in the *Xenopus* oocyte and challenged with a large panel of human odors (Liu et al., 2017a). In general, ORs with strong responses were tuned to aldehydes, ketones, alcohols, and aromatic compounds. Functional tests of these ORs in response to the components of aggregation pheromone also revealed that most of these components were encoded by multiple ORs with various tuning properties (Liu et al., 2017b). In addition, three ORs were identified as potent DEET receptors, even though DEET is not very effective in repelling bed bugs. Interestingly, these DEET-sensitive ORs presented even higher sensitivity to certain botanical terpenes/terpenoids that generally displayed much stronger repellency for bed bugs than DEET (Liu et al., 2017c).

#### Ionotropic Receptors and Gustatory Receptors

Ionotropic glutamate receptors (iGluRs) are chemosensory receptors that mediate neuronal communication between synapses in both vertebrate and invertebrate nervous systems. They comprise one of the three superfamilies used to classify IRs based on their predicted molecular structures, including an extracellular N-terminus, a cytoplasmic C-terminus, a bipartite ligand-binding domain, and an ion channel. However, IRs differ from the well-documented kainate, α-amino-3-hydroxy-5-menthyl-isoxazole-4-propionate (AMPA), or N-menthyl-D-aspartate (NMDA) classes of iGluRs as they (1) lack the characteristic glutamate interacting residues but instead have divergent ligand-binding domains; and (2) accumulate in sensory dendrites rather than at synapses (Benton et al., 2009). Phylogenetic studies have revealed that IRs are conserved across bacteria, plants, and animals, which suggests an evolutionarily ancient function in chemosensation (Benton et al., 2009). IRs in coeloconic OSNs are known to be responsible for detecting organic acids, amines, and polyamines (Benton et al., 2009; Ai et al., 2010; Hussain et al., 2016). Like Orco, IR8a, IR25a, and IR76b are highly conserved across different species and are considered to function as co-receptors with other IRs in mediating the olfactory responses to semiochemicals (Croset et al., 2010). For example, in *D. melanogaster*, IR64a and IR8a are physically associated in the OSNs and constitute a functional channel when co-expressed *in vitro* in *Xenopus* oocytes (Ai et al., 2013). In *An. gambiae*, both *IR25a* and *IR76b* are required for the functional expression of *IR41a* and *IR41c* in *Xenopus* oocytes, while IR8a is needed for the expression of *IR75k* in oocytes (Pitts et al., 2017). In addition to its role as a co-receptor, *Drosophila* IR25a has been shown to function as a thermosensor as well as playing a role in establishing the insect’s circadian rhythm (Chen et al., 2015), suggesting other potentially important functions of IRs in insect physiology.

In the kissing bug, *R. prolixus,* these three IR co-receptors (IRCO) genes (*IR8a*, *25a*, and *76b*) have been investigated to determine their expression patterns under different physiological and developmental conditions. IRCOs are known to be transcribed in the antennae of all nymph instar development stages and in both male and female kissing bugs (Latorre-Estivalis et al., 2016) and all three of these IRCOs are down-regulated by blood-feeding and up-regulated after the imaginal molt (Latorre-Estivalis et al., 2015), which underlines the plasticity of triatomine olfactory-mediated behaviors. In addition to the IRCOs, the expression patterns for 15 *R. prolixus* IRs in different tissues or sexual conditions have been characterized. Although most (11 out of 15) of these RproIRs were expressed in the antennae of all developmental instars, some exceptions have been reported. For example, no *RproIR75e* expression was observed in embryos and *RproIR20a* was not detected in first instar nymphs; neither *RproIR103* nor *RproIR104* were found in the antennae in either the nymph instars or adults of either sex (Latorre-Estivalis et al., 2016).

Based on the genomic data, 33 and 30 IRs have been annotated in *R. prolixus* and *C. lectularius*, respectively (Figures 1D). Functional studies of *Drosophila* IRs have suggested that organic acids and amine compounds are likely to be the primary ligands for IRs (Benton et al., 2009; Ai et al., 2010). Given that the C type sensilla in bed bugs show extreme sensitivity to amine compounds (Liu and Liu, 2015), certain IRs may be expressed in these sensilla. In the kissing bug, *R. prolixus*, ammonia and amines from vertebrate excretion were found to induce an obvious attraction response, suggesting that some factors in the kissing bug olfactory system (e.g. IRs) are actively sensing these compounds and guiding the host-searching behavior (Otálora-Luna and Guerin, 2014). However, as yet none of the IRs from either kissing bugs or bed bugs have been functionally characterized, further studies on these IRs are therefore necessary to clarify the response profiles of IRs in both insects.

In addition to ORs and IRs, GRs are involved in food searching and feeding stimulation. GRs are known to be responsible for detecting CO_2_, amines, and polyamines, and compounds in food sources including sugars, bitter tastes, and toxins (Liman et al., 2014; MacWilliam et al., 2018). Based on their genome sequences, there are 36 and 31 GRs in *C. lectularius* and *R. prolixus* (Figures 1D, 3), respectively. Among these, no sugar receptors have been identified in either bed bugs (Benoit et al., 2016) or kissing bugs (Mesquita et al., 2015), which explains the lack of phagostimulation by glucose in *C. lectularius* (Romero and Schal, 2014). This lack of sugar receptors has also been documented in other obligate blood-feeders, including tsetse flies (Obiero et al., 2014) and lice (Kirkness et al., 2010). Interestingly, the CO_2_ sensory GR subfamily is absent in *R. prolixus*, while the four putative CO_2_ sensory GRs that have been identified in bed bugs are phylogenetically conserved with the CO_2_ receptors in flies, moths, beetles, and one termite species (Terrapon et al., 2014). Future endeavors to investigate the response profiles of GRs from either kissing bugs or bed bugs would thus advance our understanding of chemoreception in both insects considerably.

### Chemosensation-Based Applications

**Figure 4:**
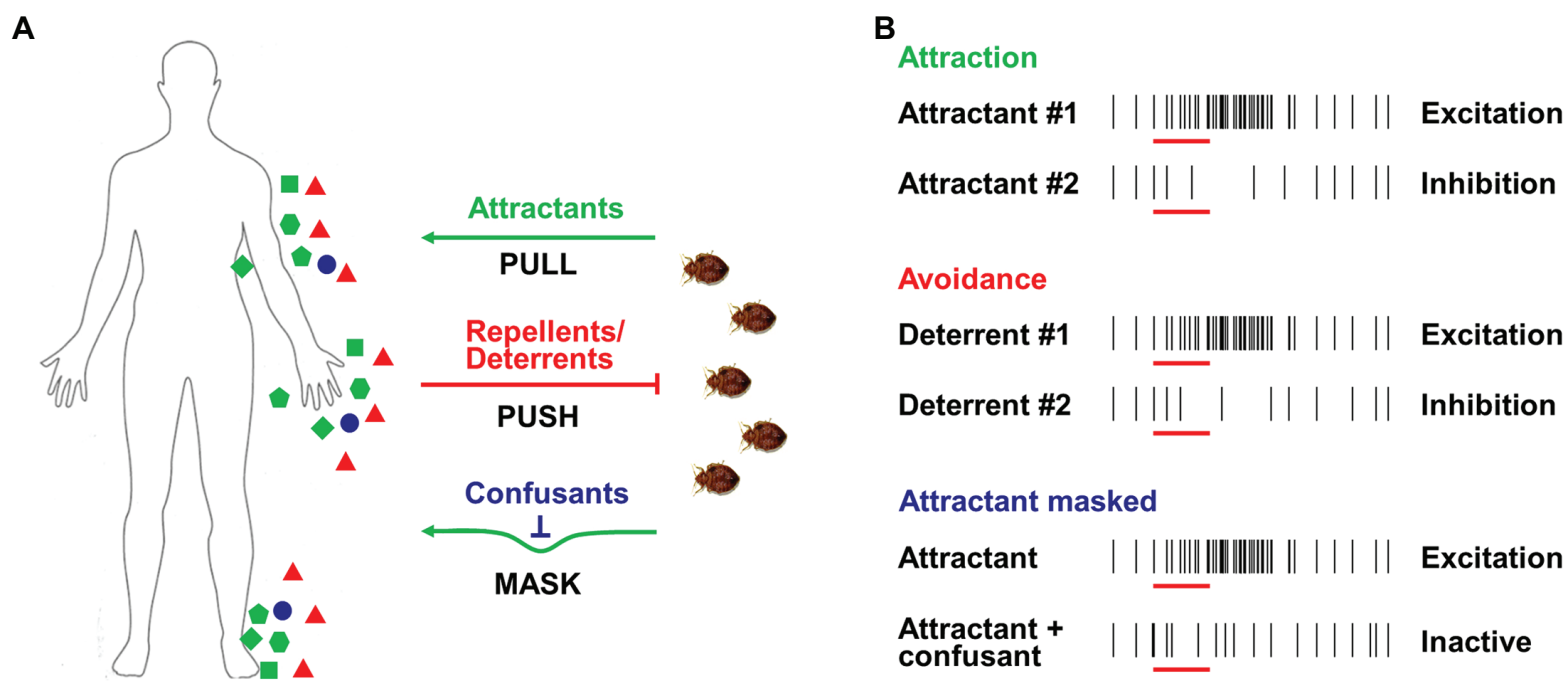
Push-pull strategies and odor-evoked excitation/inhibition activity of ORNs. **(A)** Push-pull or stimulo-deterrent diversionary (SDD) strategies used for insect control; green dots indicate attractants from hosts (PULL), red dots indicate repellents/deterrents (PUSH), and blue dots indicate confusants (MASK). **(B)** Excitation or inhibition activities of ORNs caused by attractants, repellents/deterrents and confusants.

Due to the biting nuisance and risk of potential disease transmission, the effective management of both kissing bugs and bed bugs is one of the basic aims of research in this area and a long-term goal for scientists (Boase and Naylor, 2014; Zermoglio et al., 2015). Various strategies have been applied in the battle to control these two pests, with many based on the widespread use of insecticides. However, the intense application of insecticides leads to strong selection pressure, building up resistance in insect populations and dramatically impairing the efficiency of insecticides. Therefore, new approaches are continually being explored as a matter of urgency. Several promising approaches, such as push-pull or stimulo-deterrent diversionary (SDD) strategies (Cook et al., 2007; Figure 4A), are based on the latest research on insect chemosensation. Insects such as bed bugs and kissing bugs can be attracted by host odors (PULL) or repelled by repellents/deterrents (PUSH), while another option is to mask host odors using confusants (MASK). These novel approaches using chemicals, attractants, repellents, and/or confusants are expected to contribute to reducing the vector borne disease transmission.

#### Chemical Lures

As one of the most important cues released from human skin and breath, CO_2_ is highly attractive to most hematophagous insects, including both kissing bugs and bed bugs (Barrozo and Lazzari, 2004; Wang et al., 2009; Singh et al., 2013; Indacochea et al., 2017). It is therefore not surprising that CO_2_ has been extensively incorporated in many of the bed bug traps that are commercially available as it displays high efficiency in terms of bug catches. Multiple host-related odorants that are generally added to the bait also exert a synergistic effect in attracting kissing bugs or bed bugs. For example, 1-octen-3-ol and nonanal, which are identified in human emanation (Bernier et al., 2000) and bed bug aggregation pheromone (Weeks et al., 2020), display strong activation on OSNs and ORs of bed bugs (Liu and Liu, 2015; Liu et al., 2017a). In laboratory two-choice behavioral assays, 1-octen-3-ol and nonanal have both been shown to attract bed bugs (Singh et al., 2012; Figure 4B). When a chemical mixture containing both 1-octen-3-ol and nonanal is combined with CO_2_ in a bed bug trap, a synergistic effect has been reported, with increased trap catches (Singh et al., 2012). Since a large number of human odorants have been successively examined in bed bugs using electrophysiological approach and some of them elicit strong neuronal responses in different types of olfactory sensilla (Liu and Liu, 2015), future behavioral studies under laboratory conditions or in the field should identify some promising candidates with potent attraction for bed bugs.

In the kissing bug *T. infestans*, ammonia is reported to activate two types of grooved peg sensilla, making it a strong attractant for *T. infestans* (Taneja and Guerin, 1997). Another study found that a carbon dioxide-free attractant containing three human odorants (ammonia, L-(+)-lactic acid, and hexanoic acid) significantly increased bug catches for both *R. prolixus* and *T. infestans* (Guidobaldi and Guerenstein, 2013). In addition, male *R. prolixus* is attracted by a synthetic female-pheromone blend comprised of ten compounds, which elicit neuronal response from basiconic olfactory sensillum (Bohman et al., 2018). Taken together, these behavioral assays indicate the potential utility of using human odors or pheromone components to boost the performance of chemical lures for both bed bugs and kissing bugs, as well as highlighting the need to explore the potency of other OSNs/receptor-sensitive human odors.

#### Chemical Repellents/Confusants

Several different mechanisms have been proposed for chemical repellency in insect olfaction. First, certain compounds are known to block the attractant binding sites of OBP(s), resulting in insensitivity or reduced sensitivity to attractants. For example, it has been reported that in the mosquito *An. gambiae,* the synthetic repellent DEET and two natural repellents, 6-menthyl-5-heptene-2-one and eugenyl acetate, occupy the active binding site of OBP1, which is thought to be critical in preventing the transportation of some key attractant odorants (Murphy et al., 2012; Tsitsanou et al., 2012; Affonso et al., 2013). Second, some odorants may cause avoidance behavior by activating or inhibiting the ORN activities in insects (Figure 4B). For instance, in the mosquito *C. quinquefasciatus*, DEET is known to activate OR136b, triggering an aversive response (Xu et al., 2014); geraniol has also been shown to inhibit the activity of *Or10a*-ORN and *Or42b*-ORN and induce avoidance behavior in *Drosophila* (Cao et al., 2017). Third, some odorants may block/inhibit (or mask) the excitatory responses elicited by attractive compounds or alter the temporal structure of the insect’s ORN response to attractants (Figure 4B). Ditzen et al. (2008) reported that DEET significantly blocks the neuronal response of *An. gambiae* to one human odorant (1-octen-3-ol), while Pellegrino et al. (2011) found that DEET appears to scramble the olfactory responses of *D. melanogaster* to some odors, although the precise mechanism is unclear. Bohbot et al. (2011) also suggested that DEET significantly inhibits the function of *Aedes* mosquitos’ ORs in response to ligands. Odorants that alter the temporal structure of the ORN response may also affect insect olfaction-mediated behavior. It has been reported that in either mosquito or moth, some mimicking compounds evoke continual firing in the primary compound-sensitive ORN, which disrupts the normal attractive behavioral response of those insects (Kramer, 1992; Turner et al., 2011).

In the common bed bug, certain ORNs and ORs were found to be directly activated by DEET while DEET also blocked the excitatory responses of ORNs and ORs to some human odors as well as manipulating the temporal dynamic of the odor-evoked neuronal response, which may result in the significant repellency of DEET agaist the bed bugs (Liu et al., 2017c). The same study also identified some components from essential oils, such as (+)-menthone, linalyl acetate and menthyl acetate, which effectively activated multiple ORNs and ORs and elicited very potent repellency against the bed bugs with a corresponding dose threshold of 10–100 fold lower than that of DEET (Liu et al., 2017c). In *R. prolixus*, one male-enriched OR (RproOR80) was functionally sensitized to 4-methylcyclohexanol, which turned out to be a strong repellent for kissing bugs by inducing a significant decrease in residence time to the host and a remarkable reduction in blood intake (Franco et al., 2018). This reverse chemical ecology strategy has also been adopted for identifying compounds with biological significance for other blood-feeding insects (Leal et al., 2008; Choo et al., 2018), agricultural insects (Xu et al., 2021), and mammals (Zhu et al., 2017). All these studies highlight the value of conducting further explorations of novel behaviorally active semiochemicals based on the reverse chemical ecology strategy for better controlling insect pests, such as bed bugs and kissing bugs and terminating the potential disease transmission.

## Author Contributions

NL, FL, ZC, and YZ: conceived and designed the study, wrote thepaper and reviewed the manuscript. All authors contributed to the article and approved the submitted version.

## Conflict of Interest

The authors declare that the research was conducted in the absence of any commercial or financial relationships that could be construed as a potential conflict of interest.

## Publisher’s Note

All claims expressed in this article are solely those of the authors and do not necessarily represent those of their affiliated organizations, or those of the publisher, the editors and the reviewers. Any product that may be evaluated in this article, or claim that may be made by its manufacturer, is not guaranteed or endorsed by the publisher.
